# Development and validation of a risk prediction model for postoperative frailty in breast cancer patients

**DOI:** 10.7717/peerj.21733

**Published:** 2026-09-22

**Authors:** Ye Zhou, Lili Li, Lin Jiang, Lihua Zhou, Huan Qiu

**Affiliations:** 1 School of Nursing, Anhui Medical University, Hefei, Anhui, China; 2 Department of Breast Surgery, The First Affiliated Hospital of USTC, Division of Life Sciences and Medicine, University of Science and Technology of China, Hefei, Anhui, China; 3 Department of Nursing, The First Affiliated Hospital of Anhui Medical University, Hefei, Anhui, China

**Keywords:** Breast cancer, Postoperative frailty, Risk factors, Predictive model

## Abstract

**Background:**

Patients who have undergone breast cancer (BC) surgery are a high-risk population for frailty. This study aims to analyze risk factors for frailty in these patients, develop a risk prediction model, and validate its predictive performance, thereby providing a reference for frailty prevention in postoperative breast cancer patients.

**Methods:**

A total of 286 BC patients who underwent surgical treatment at the Department of Breast Surgery, the First Affiliated Hospital of University of Science and Technology of China (USTC), between October 2024 and March 2025, participated in a cross-sectional study and were surveyed using a general information questionnaire, the Chinese version of the Tilburg Frailty Indicator (TFI), and the Pittsburgh Sleep Quality Index (PSQI). We used logistic regression to analyze factors influencing postoperative frailty in BC patients and constructed a risk prediction nomogram. We evaluated the model’s predictive performance using receiver operating characteristic (ROC) curves, calibration curves, the Hosmer-Lemeshow test, and decision curve analysis (DCA), with internal validation conducted *via* 10-fold cross-validation.

**Results:**

The incidence of postoperative frailty among BC patients was 29.37%. Logistic regression analysis identified the following factors as significant influences on frailty in this population (*P* < 0.05): a history of diabetes, poor sleep quality, having children as the primary caregivers, urban employee basic medical insurance, higher cholesterol levels, higher body mass index (BMI), higher exercise frequency, and undergoing breast conserving surgery. The results of the Hosmer–Lemeshow test showed that *χ*^2^ = 4.436, *P* = 0.816. The model showed an area under the ROC curve was 0.813 after internal validation.

**Conclusion:**

The created prediction model provided a precise, individualized evaluation of postoperative frailty risk in BC patients. It can be used to identify individuals at high risk of postoperative frailty in BC patients and to guide healthcare professionals in promptly implementing targeted interventions.

## Introduction

Breast cancer (BC) is the most common malignant tumor among women worldwide. According to data from the International Agency for Research on Cancer in 2022 (Bray et al., 2024), there were 2.3 million new cases of BC worldwide, representing 23.8% of all female cancers and 15.4% of female cancer-related deaths. Surgery plays a central role in the treatment strategies for BC, with chemotherapy, radiotherapy, targeted therapy, and endocrine therapy serving as adjunctive modalities (Goede, 2023). However, although these treatments effectively control tumor progression, they may also induce frailty in patients with BC. Frailty represents a state of physiological, psychological, and social vulnerability, characterized by a decline in function across multiple physiological systems, with an increased vulnerability to stressors (Dent et al., 2019; Jin et al., 2021). This condition can lead to a series of negative health outcomes, including extended hospital stays, increased healthcare costs, and diminished quality of life (Tsai et al., 2022). Studies indicate that approximately 43% of BC patients experience symptoms of frailty (Wang et al., 2022). Cancer itself, surgery, and adjuvant therapies trigger multi-system pathophysiological changes that increase vulnerability and blunt stress tolerance in BC patients, making these patients more prone to frailty (Wang et al., 2024). In addition, psychosocial factors such as depression, anxiety, and insufficient social support may further worsen frailty in BC patients (Bu et al., 2023).

Current research on frailty in BC patients has predominantly concentrated on the frailty status of elderly and preoperative populations (Fred, 2019; Lu et al., 2023). However, after surgery, BC patients not only confront the physical trauma of the operation but also face the psychological impact of losing a secondary female sexual characteristic (Khajoei et al., 2023). Therefore, the risk of postoperative frailty in BC patients deserves clinical attention. Notably, frailty is a dynamic process. By developing a predictive model for postoperative frailty risk in BC patients, we can identify risk factors at an early stage and implement timely interventions. These can effectively prevent the occurrence of frailty, mitigate adverse clinical outcomes, and promote recovery in BC patients (Jin et al., 2021). In summary, BC patients are at high risk of frailty. Early detection and identification of frailty are crucial for the management and treatment of these patients. This study aimed to identify risk factors for postoperative frailty in BC patients, develop a risk prediction model, and evaluate its predictive performance. The goal is to accurately predict the risk of postoperative frailty in BC patients at an early stage, thereby providing references for clinical healthcare professionals in the prevention and management of frailty in this patient population.

## Materials and Methods

### Participants

In this study, a convenience sampling method was employed to recruit a total of 286 BC patients from the First Affiliated Hospital of the University of Science and Technology of China (USTC) between October 2024 and March 2025. Questionnaires were administered to the patients 3 to 5 days post-surgery. The inclusion criteria were as follows: (1) participants aged 18 years or older; (2) pathologically confirmed breast cancer following surgery; (3) participants with all types and stages of BC were included in the study; and (4) voluntary participation in the study. The exclusion criteria included: (1) impaired consciousness, communication disorders, or reading difficulties; (2) a history of mental illness; and (3) comorbidity with other tumors or the presence of distant metastasis. This study received approval from the Ethics Committee of the First Affiliated Hospital of USTC (Approval No. 204KY-484), and all participants provided written informed consent.

Sample Size Calculation: The sample size estimation for this study was based on the methodology for multivariate logistic regression models (Peduzzi et al., 1996), which recommends that the required sample size should be 5 to 10 times the number of independent variables. Given that this study included 32 independent variables, the calculated sample size ranged from 160 to 320 cases. To account for a potential 20% rate of invalid questionnaires, the final required sample size was adjusted to between 192 and 384 cases. A total of 300 questionnaires were distributed. After excluding 14 invalid responses, 286 valid questionnaires were recovered, resulting in an effective response rate of 95%.

### Measurement

#### General information questionnaire for BC patients

The researchers developed this questionnaire through a systematic literature review and discussions with experts. It consists of four sections: (1) Demographic Information: gender, age, marital status, educational level, occupation, type of medical insurance, primary caregiver, monthly household income per capita, body mass index (BMI), and exercise frequency; (2) Disease-Related Information: comorbid chronic diseases, chemotherapy history, gastrointestinal reactions, and oral mucositis; (3) Laboratory Indicators: total protein, albumin, hemoglobin, and total cholesterol; and (4) Surgery-Related Information: surgical method and pathological type.

#### The Chinese version of the Tilburg Frailty Indicator (TFI)

The TFI was originally developed by Gobbens et al. (2010) in 2001 and later sinicized by Xi, Guo & Sun (2013). It is a comprehensive frailty assessment tool covering physical, psychological, and social multidimensional evaluations, which can comprehensively, objectively, and accurately reflect the overall frailty status of patients (Zamora-Sánchez et al., 2022). The TFI consists of three dimensions and 15 items: physical dimension (weight loss, health status, walking difficulties, balance ability, vision, hearing, grip strength, and fatigue), psychological dimension (memory, anxiety, depression, and coping ability), social dimension (living alone, social relationships, and social support). Among the 15 items, 11 are scored using a binary “Yes/No” scale, and four are scored using a three-point “Yes/Sometimes/No” scale. The total score ranges from 0 to 15, with a score of ≥ 5 indicating frailty; a higher score indicates a more severe degree of frailty. The Cronbach’s α coefficient of the Chinese Version of the TFI is 0.747 (Song et al., 2020). The authors received permission to use this instrument from the copyright holders.

#### Pittsburgh sleep quality index (PSQI)

The Pittsburgh Sleep Quality Index (PSQI) was originally developed by Buysse et al. (1989) and later sinicized by Liu et al. (1996) to evaluate patients’ sleep quality over the past month. The scale consists of 19 self-reported items and five observer-reported items. Among these, the 19th self-reported item and the five observer-reported items are not included in scoring. The 18 items that contribute to the score are divided into seven dimensions: sleep quality (scored based on item 6); sleep latency (total score of items 2 and 5a); sleep duration (scored based on item 4); sleep efficiency (calculated using responses from items 1, 3, and 4); sleep disturbances (total score of items 5b to 5j); use of sleep medication (scored based on item 7); daytime dysfunction (total score of items 8 and 9).

Each dimension is converted to a score ranging from 0 to 3 based on the sum of its corresponding items. The total score ranges from 0 to 21, with higher scores indicating poorer sleep quality (Liao et al., 2022). The specific criteria are as follows: a score of 0–5 indicates good sleep quality, 6–10 indicates poor sleep quality, and 11–21 indicates very poor sleep quality. The Cronbach’s α coefficient of the Chinese Version of the PSQI is 0.842 (Liu et al., 1996). The authors received permission to use this instrument from the copyright holders.

### Data collection and quality control

Before the survey, investigators received standardized training. During the survey, uniform instructions were used, and investigators provided face-to-face guidance to patients to complete the online questionnaire *via* Wenjuanxing (a professional online survey platform in China). For patients who had difficulty completing the questionnaire independently, investigators assisted them using non-leading explanatory language. Data export and analysis were jointly conducted by two researchers, who also performed item-by-item verification to ensure data accuracy and completeness. Strict quality control measures were implemented throughout the study, including the planning, implementation, data collection, and analysis phases.

### Statistical analysis

Statistical analyses were performed using the statistical software SPSS 24.0 and R software (version 4.1.2). Descriptive statistics: categorical data were presented as n (%), and comparisons between groups were conducted using the chi-square test. For continuous data that conformed to a normal distribution, they were expressed as (mean ± standard deviation), and inter-group comparisons were performed using the independent samples *t*-test. Influencing factor analysis: Least Absolute Shrinkage and Selection Operator (LASSO) regression was used for preliminary variable screening and dimensionality reduction of postoperative frailty in BC patients. The optimal *λ* value was determined by 10-fold cross-validation, and all variables with non-zero regression coefficients were retained. All screened variables were entered into the multivariable logistic regression model, and variables with *P* < 0.05 were retained as final independent predictors, and a nomogram was constructed. Model evaluation: The receiver operating characteristic (ROC) curve was used to evaluate the discriminative ability of the nomogram. Calibration curves and the Hosmer-Lemeshow test were used to assess its calibration. Decision curve analysis (DCA) was conducted to evaluate its clinical applicability. Finally, 10-fold cross-validation was used for the internal validation of the nomogram.

## Results

### Comparison of general data between the two groups

A total of 286 postoperative BC patients were included in this study. Using a cutoff score of ≥5 on the TFI as the criterion, patients were divided into the frailty group (*n* = 84) and the non-frailty group (*n* = 202). The incidence of frailty was 29.37%. Statistically significant differences were observed between the two groups in terms of age, total cholesterol level, type of medical insurance, history of diabetes, surgical method, and PSQI scores (*P* < 0.05). Details are shown in Table 1.

**Table 1:** Comparison of general patient characteristics between the two groups.

Variables	Total (*n* = 286)	Non-frailty group (*n* = 202)	Frailty group (*n* = 84)	Statistic	*P*-value
Age (years), Mean ± SD	54.05 ± 11.33	52.96 ± 11.50	56.68 ± 10.51	*t* = − 2.552	0.011
Total protein (g/L), Mean ± SD	65.46 ± 5.84	65.31 ± 5.71	65.83 ± 6.16	*t* = − 0.686	0.493
Albumin (g/L), Mean ± SD	39.41 ± 3.19	39.27 ± 3.05	39.75 ± 3.51	*t* = − 1.139	0.256
Hemoglobin (g/L), Mean ± SD	118.14 ± 15.43	118.09 ± 13.82	118.26 ± 18.85	*t* = − 0.082	0.935
Total cholesterol (mmol/L), Mean ± SD	4.74 ± 0.94	4.83 ± 0.98	4.52 ± 0.81	*t* = 2.623	0.009
BMI (kg/m^2^), Mean ± SD	24.56 ± 3.55	24.76 ± 3.48	24.09 ± 3.70	*t* = 1.462	0.145
Marital status, n (%)				*χ*^2^ = 2.697	0.260
Unmarried	5 (1.75)	4 (1.98)	1 (1.19)		
Married	261 (91.26)	187 (92.57)	74 (88.10)		
Divorced/Widowed	20 (6.99)	11 (5.45)	9 (10.71)		
Primary caregiver, n (%)				*χ*^2^ = 3.389	0.335
Spouse	189 (66.08)	129 (63.86)	60 (71.43)		
Parents	17 (5.94)	15 (7.43)	2 (2.38)		
Children	61 (21.33)	45 (22.28)	16 (19.05)		
Other	19 (6.64)	13 (6.44)	6 (7.14)		
Education level, n (%)				*χ*^2^ = 1.935	0.380
Primary school and below	94 (32.87)	62 (30.69)	32 (38.10)		
Junior high school/Senior high school	133 (46.50)	95 (47.03)	38 (45.24)		
College and above	59 (20.63)	45 (22.28)	14 (16.67)		
Occupation, n (%)				*χ*^2^ = 4.580	0.101
Employed	65 (22.73)	52 (25.74)	13 (15.48)		
Retirement	74 (25.87)	47 (23.27)	27 (32.14)		
Unemployed or otherwise	147 (51.40)	103 (50.99)	44 (52.38)		
Medicare types, n (%)				*χ*^2^ = 6.572	0.010
Resident medical insurance	166 (58.04)	107 (52.97)	59 (70.24)		
Employee medical insurance	120 (41.96)	95 (47.03)	25 (29.76)		
Residence, n (%)				*χ*^2^ = 2.151	0.143
Rural areas	109 (38.11)	71 (35.15)	38 (45.24)		
Towns	177 (61.89)	131 (64.85)	46 (54.76)		
Monthly household income per capita, n (%)				*χ*^2^ = 1.396	0.497
<1,000 yuan	43 (15.03)	28 (13.86)	15 (17.86)		
1,000–5,000 yuan	178 (62.24)	130 (64.36)	48 (57.14)		
>5,000 yuan	65 (22.73)	44 (21.78)	21 (25.00)		
Complicated with diabetes, n (%)				*χ*^2^ = 14.051	<0.001
No	254 (88.81)	189 (93.56)	65 (77.38)		
Yes	32 (11.19)	13 (6.44)	19 (22.62)		
Complicated with hypertension, n (%)				*χ*^2^ = 2.450	0.118
No	223 (77.97)	163 (80.69)	60 (71.43)		
Yes	63 (22.03)	39 (19.31)	24 (28.57)		
Complicated with cerebral infarction, n (%)				*χ*^2^ = 3.248	0.072
No	271 (94.76)	195 (96.53)	76 (90.48)		
Yes	15 (5.24)	7 (3.47)	8 (9.52)		
Surgical method, n (%)				*χ*^2^ = 8.492	0.014
Modified Radical Mastectomy	209 (73.08)	138 (68.32)	71 (84.52)		
Breast-Conserving Surgery	50 (17.48)	43 (21.29)	7 (8.33)		
Implant-Based Reconstruction	27 (9.44)	21 (10.40)	6 (7.14)		
Pathological Type, n (%)				*χ*^2^ = 1.389	0.499
Carcinoma *in situ*	64 (22.38)	43 (21.29)	21 (25.00)		
Invasive carcinoma	218 (76.22)	157 (77.72)	61 (72.62)		
Low/undifferentiated carcinoma	4 (1.40)	2 (0.99)	2 (2.38)		
Neoadjuvant chemotherapy, n (%)				*χ*^2^ = 1.608	0.205
No	220 (76.92)	160 (79.21)	60 (71.43)		
Yes	66 (23.08)	42 (20.79)	24 (28.57)		
Postoperative gastrointestinal reaction, n (%)				*χ*^2^ = 0.163	0.686
No	222 (77.62)	155 (76.73)	67 (79.76)		
Yes	64 (22.38)	47 (23.27)	17 (20.24)		
Oral mucositis, n (%)				*χ*^2^ = 3.407	0.065
No	266 (93.01)	192 (95.05)	74 (88.10)		
Yes	20 (6.99)	10 (4.95)	10 (11.90)		
Exercise Frequency, n (%)				*χ*^2^ = 4.770	0.092
None	147 (51.40)	96 (47.52)	51 (60.71)		
<3 times/week	71 (24.83)	52 (25.74)	19 (22.62)		
≥3 times/week	68 (23.78)	54 (26.73)	14 (16.67)		
PSQI, n (%)				*χ*^2^ = 29.107	<0.001
Good sleep quality	139 (48.60)	116 (57.43)	23 (27.38)		
Moderately poor quality	137 (47.90)	84 (41.58)	53 (63.10)		
Severely poor quality	10 (3.50)	2 (0.99)	8 (9.52)		

**Notes.**

t *t*-test *χ*^2^ Chi-square test SD standard deviation

### Screening of characteristic variables for postoperative frailty in BC patients

**Figure 1:**
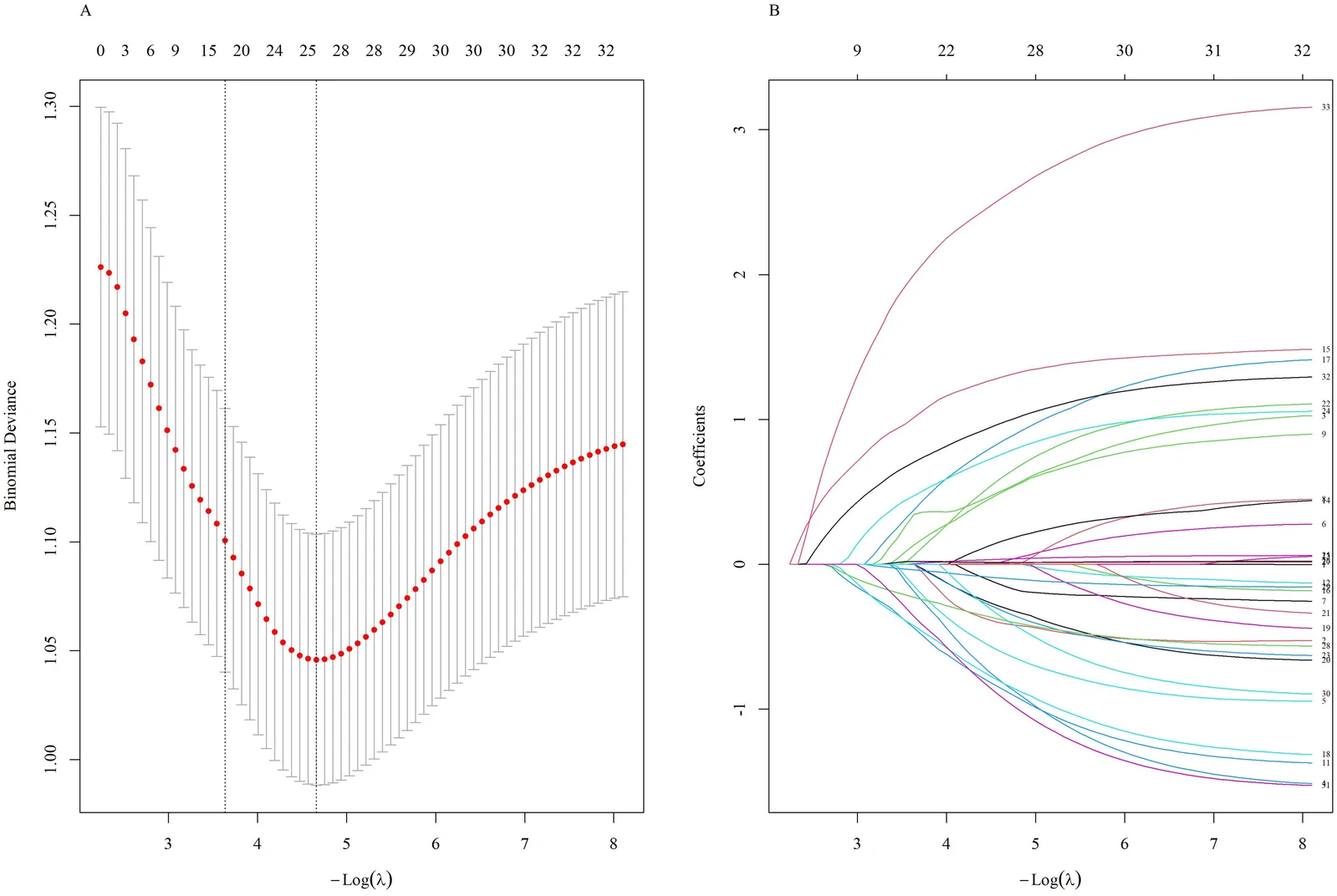
Results of LASSO regression analysis. (A) Plot of binomial deviance based on 10-fold cross-validation. The horizontal axis represents −log(*λ*), and the vertical axis represents binomial deviance. The two vertical dashed lines correspond to *λ*_min_ (lambda with the minimum deviance) and *λ*_1se_ (lambda under the one-standard-error rule), respectively. (B) LASSO regression coefficient path plot. The horizontal axis denotes −log(*λ*), and the vertical axis represents regression coefficients of variables. Each curve illustrates the trajectory of the regression coefficient for an independent variable as *λ* varies. A total of 16 variables with non-zero regression coefficients were screened according to the criterion of *λ*_1se_ = 0.02638044 and included in the subsequent model construction.

Given the large number of independent variables included in this study, LASSO regression was used for dimensionality reduction (Luo et al., 2023) to conduct preliminary variable screening. A 10-fold cross-validation was applied, and the maximum *λ* value within one standard error of the minimum cross-validation error (*λ*1se) was selected as the optimal penalty parameter for the LASSO model. Variables with non-zero regression coefficients at this *λ* value were retained for further analysis. The LASSO regression results showed that the tuning parameter *λ* at one standard error (*λ*1se) was 0.02638044 (see Fig. 1), corresponding to 16 variables with non-zero regression coefficients. These variables were: marital status, primary caregiver, occupation, type of medical insurance, diabetes, history of cerebral infarction, surgical method, history of chemotherapy, gastrointestinal reactions, oral mucositis, total protein, albumin, total cholesterol, BMI, exercise frequency, and PSQI score. These 16 variables were identified as characteristic predictors of postoperative frailty in BC patients and were all included in the subsequent multivariate logistic regression analysis (see Fig. 1).

### Multivariate logistic regression analysis of postoperative frailty in BC patients

The presence or absence of postoperative frailty was defined as the dependent variable, while the 16 characteristic variables initially screened by LASSO regression were included as independent variables in the multivariate logistic regression equation. Variables with *P* < 0.05 were retained as final predictors. Categorical variables were converted into dummy variables for the regression analysis. Continuous variables were included in the model using their original measured values. The results of the multivariate logistic regression analysis revealed that (see Table 2) a history of diabetes (odds ratio (OR) = 5.24, 95% confidence interval (CI) [1.97–13.94]) and poor sleep quality (moderately poor sleep: OR = 3.66, 95% CI [1.82–7.33]; severely poor sleep: OR = 25.29, 95% CI [3.97–161.16]) were risk factors for postoperative frailty in BC patients. Higher total cholesterol (OR = 0.60, 95% CI [0.41–0.86]), having children as primary caregivers (OR = 0.36, 95% CI [0.14–0.89]), employee medical insurance (OR = 0.29, 95% CI [0.11–0.78]), higher BMI (OR = 0.86, 95% CI [0.77–0.95]), higher exercise frequency (≥3 times/week: OR = 0.20, 95% CI [0.08–0.52]), and undergo breast-conserving surgery (OR = 0.33, 95% CI [0.11–0.95]) were protective factors against postoperative frailty in BC patients.

**Table 2:** Multivariate logistic regression analysis results of postoperative frailty in Breast Cancer patients.

**Variables**	*β*	**S.E**	**Z**	***P***	**OR (95% CI)**
Intercept	0.393	3.133	0.125	0.900	1.48 (0.00∼687.60)
Marital status					
Unmarried					1.00 (Reference)
Married	−0.799	1.305	−0.612	0.540	0.45 (0.04∼5.81)
Divorced/Widowed	0.632	1.454	0.435	0.664	1.88 (0.11∼32.55)
Primary caregiver					
Spouse					1.00 (Reference)
Parents	−1.394	0.906	−1.538	0.124	0.25 (0.04∼1.47)
Children	−1.028	0.465	−2.210	0.027	0.36 (0.14∼0.89)
Other	0.258	0.691	0.373	0.709	1.29 (0.33∼5.01)
Occupation					
Employed					1.00 (Reference)
Retirement	0.734	0.602	1.219	0.223	2.08 (0.64∼6.78)
Unemployed or otherwise	−0.180	0.551	−0.326	0.744	0.84 (0.28∼2.46)
Medicare types					
Resident insurance					1.00 (Reference)
Employee medical insurance	−1.226	0.498	−2.462	0.014	0.29 (0.11∼0.78)
Complicated with diabetes					
No					1.00 (Reference)
Yes	1.656	0.500	3.314	0.001	5.24 (1.97∼13.94)
Complicated with cerebral infarction					
No					1.00 (Reference)
Yes	1.333	0.693	1.922	0.055	3.79 (0.97∼14.76)
Surgical method					
Modified Radical Mastectomy					1.00 (Reference)
Breast-Conserving Surgery	−1.123	0.549	−2.046	0.041	0.33 (0.11∼0.95)
Implant-Based breast Reconstruction	0.096	0.620	0.156	0.876	1.10 (0.33∼3.71)
Neoadjuvant chemotherapy					
No					1.00 (Reference)
Yes	0.824	0.451	1.826	0.068	2.28 (0.94∼5.52)
Postoperative gastrointestinal reaction					
No					1.00 (Reference)
Yes	−0.584	0.396	−1.477	0.140	0.56 (0.26∼1.21)
Oral mucositis					
No					1.00 (Reference)
Yes	1.051	0.624	1.686	0.092	2.86 (0.84∼9.71)
Total protein	0.069	0.053	1.315	0.188	1.07 (0.97∼1.19)
Albumin	0.026	0.091	0.286	0.775	1.03 (0.86∼1.23)
Total cholesterol	−0.517	0.189	−2.736	0.006	0.60 (0.41∼0.86)
BMI	−0.154	0.054	−2.833	0.005	0.86 (0.77∼0.95)
Exercise Frequency					
None					1.00 (Reference)
<3 times/week	−0.722	0.431	−1.675	0.094	0.49 (0.21∼1.13)
≥3 times/week	−1.598	0.485	−3.296	0.001	0.20 (0.08∼0.52)
PSQI					
Good sleep quality					1.00 (Reference)
Moderately poor sleep quality	1.296	0.355	3.654	0.000	3.66 (1.82∼7.33)
Severely poor sleep quality	3.230	0.945	3.419	0.001	25.29 (3.97∼161.16)

**Notes.**

OR Odds Ratio CI Confidence Interval

### Development of a nomogram prediction model for postoperative frailty risk in BC patients

**Figure 2:**
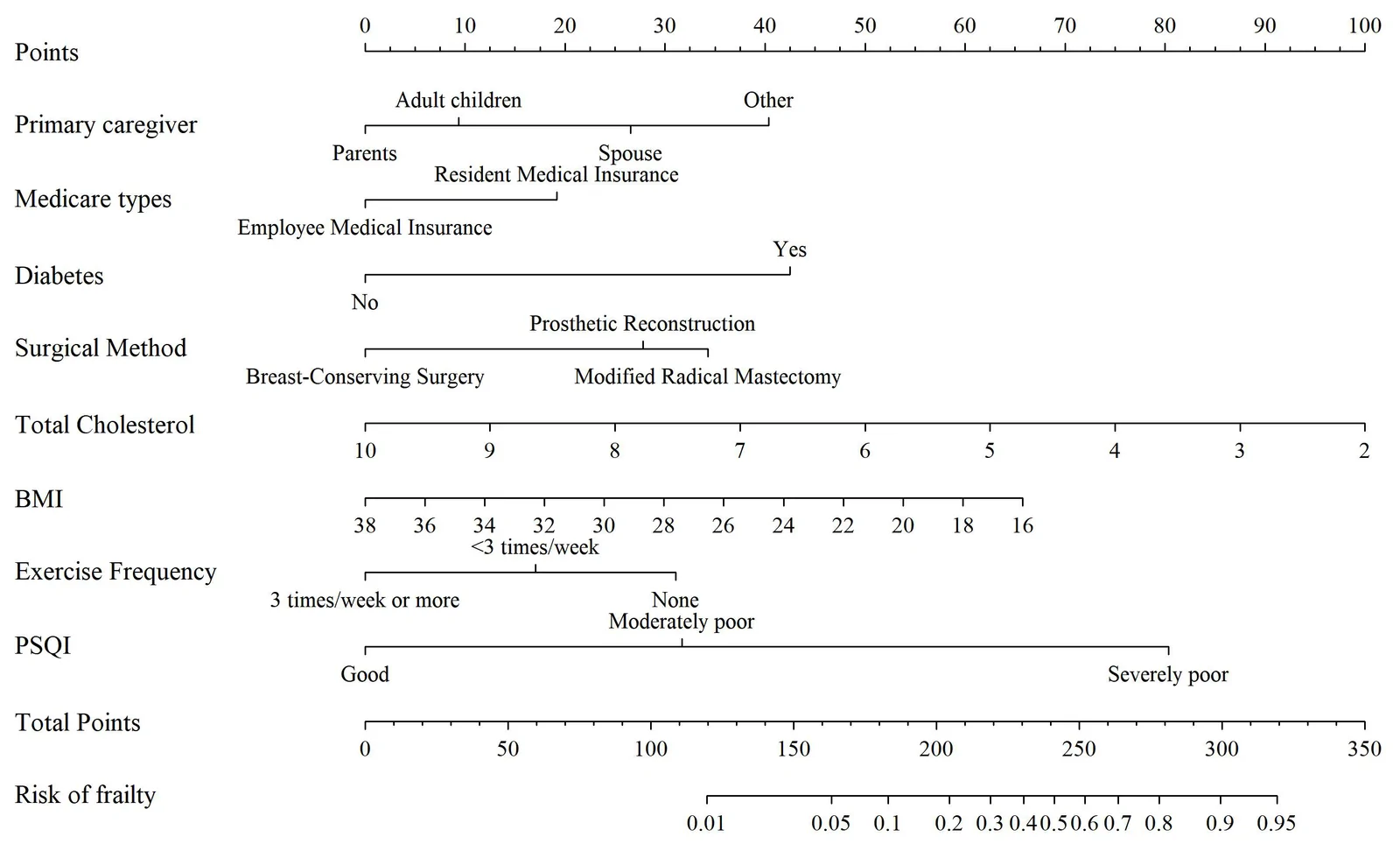
Nomogram model for the risk of postoperative frailty in breast cancer patients. Nomogram for predicting postoperative frailty risk in breast cancer patients. The model integrates eight predictors: primary caregiver, medicare type, diabetes, surgical method, total cholesterol, BMI, exercise frequency, PSQI. Instructions: (1) Draw a vertical line from each variable value to the “Score” scale to obtain the corresponding points; (2) Sum the points to get the total score; (3) Draw a vertical line from the “Total score” scale to the “Risk of frailty” scale to obtain the predicted probability. A higher total score indicates a higher risk of postoperative frailty.

A prediction model for postoperative frailty risk in BC patients was developed based on the 8 significant predictive variables identified in the logistic regression analysis, as shown in the nomogram (see Fig. 2). The model includes eight predictive variables, a score scale, a total score scale, and a frailty risk scale. Each predictive variable in the nomogram has a corresponding score on the Points scale. The steps to calculate a patient’s frailty risk are as follows: First, draw a vertical line from the measured value of each predictive variable for a patient to the Points scale to obtain the corresponding score for each variable. Then, sum the scores of all variables and find the corresponding point on the Total Points scale. Finally, draw a vertical line from this point on the Total Points scale to the Risk of Frailty scale. The value obtained represents the probability of frailty for that patient.

### Clinical applicability analysis of the nomogram prediction model

**Figure 3:**
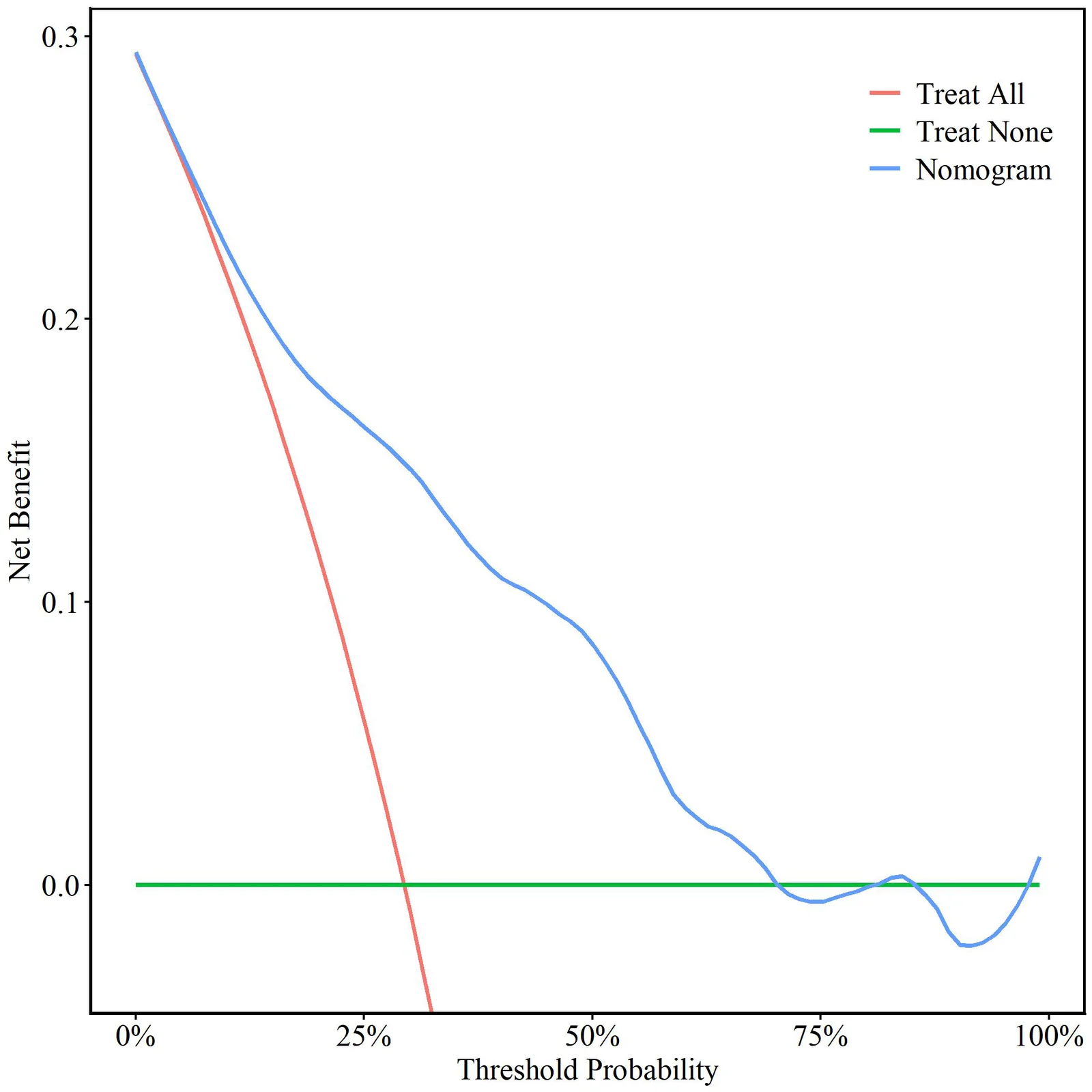
DCA curve of the nomogram model. The decision curve analysis (DCA) of the nomogram model for predicting postoperative frailty. The *x*-axis represents the threshold probability, and the *y*-axis indicates the net benefit. The horizontal green line represents the strategy of treating no patients (Treat None), the red diagonal line represents the strategy of treating all patients (Treat All), and the blue curve corresponds to the established nomogram model. Within the threshold probability ranges of 1%–69%, the net benefit curve of the nomogram outperformed the two extreme reference curves, indicating that adopting this model to guide clinical intervention could yield additional net benefit in these intervals, and the model possesses favorable clinical utility.

We performed a DCA curve (see Fig. 3) using the predicted probabilities from the logistic regression model as the test variable and the actual occurrence of postoperative frailty as the state variable. The DCA curve showed that when the threshold probability of postoperative frailty in BC patients ranged from 1% to 69%, the net benefit of applying the nomogram was significantly higher than that of the “no intervention” and “all intervention” strategies. Beyond this range (*i.e.,* below 1% or above 69%), the net benefit was close to zero or negative, suggesting that the clinical utility of the model is threshold-dependent. Therefore, the model has good clinical applicability when the estimated individual frailty risk falls within this intermediate probability interval.

### Prediction efficiency analysis and internal validation of the nomogram prediction model

**Figure 4:**
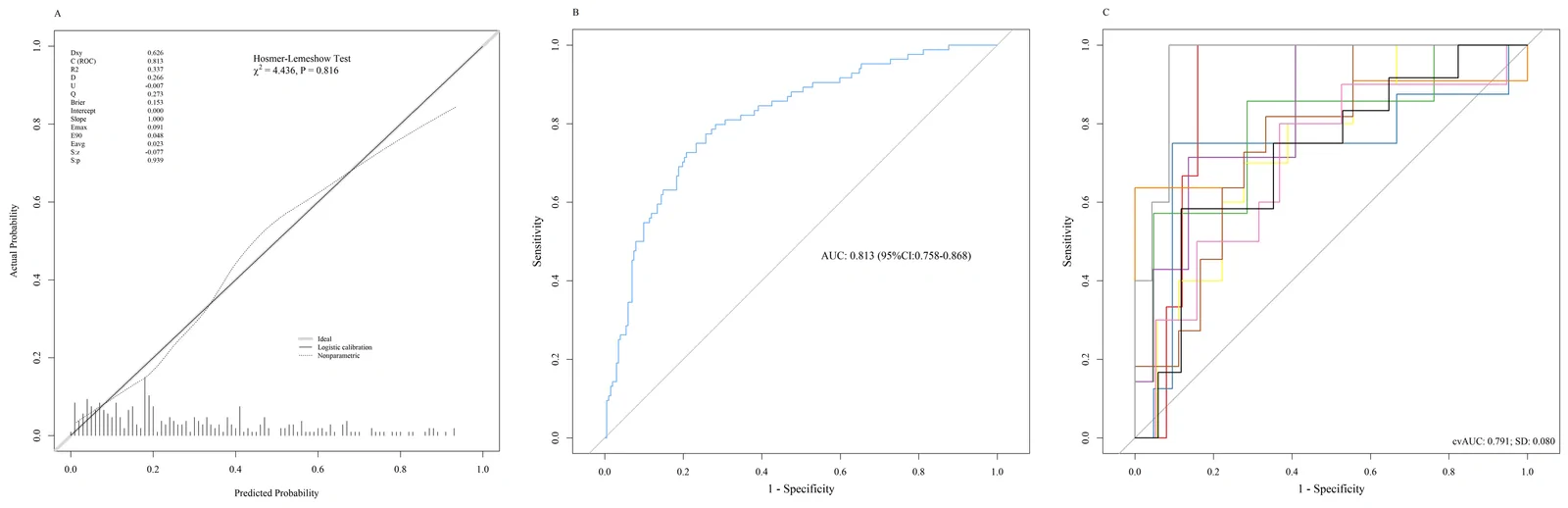
Calibration curve and Receiver operating characteristic(ROC) curve of the nomogram model. (A) Calibration curve. The solid line represents logistic calibration curve, the gray straight line is the ideal calibration line, and the dotted line denotes the nonparametric fitting curve. The Hosmer-Lemeshow test yielded (*χ*^2^ = 4.436, *P* = 0.816), and the Brier score was 0.153, indicating favorable consistency between predicted and actual probabilities and good calibration of the model. (B) Receiver operating characteristic (ROC) curve. The AUC was 0.813 (95% CI [0.758–0.868]). The optimal cutoff value was 0.327 with a Youden index of 0.518, corresponding to a sensitivity of 0.726 and specificity of 0.792, demonstrating satisfactory discrimination of the nomogram. (C) ROC curves of 10-fold cross-validation. The mean cross-validated AUC (cvAUC) was 0.791 (SD = 0.080), which verified favorable and stable discriminative performance of the model.

Calibration Evaluation: The Hosmer-Lemeshow test indicated no statistically significant difference between the predicted risk values of the model and the actual measured values (*χ*^2^ = 4.436, *P* = 0.816). The calibration curve (see Fig. 4A) showed that the risk fitting curve of the model was close to the ideal curve. The ROC curve was used to evaluate the discriminative ability of the nomogram. The area under the ROC curve (AUC) was 0.813 (95% CI [0.758–0.868]), with a specificity of 0.792, a sensitivity of 0.726, an optimal cut-off value of 0.327, and a Youden index of 0.518 (see Fig. 4B). Ten-fold cross-validation was further performed for internal validation. The results revealed that the AUC value of each fold was greater than 0.7, with a mean AUC of 0.791 (see Fig. 4C). These findings indicated that the established nomogram possessed favorable discriminatory power and excellent internal stability.

## Discussion

### Current status of postoperative frailty in BC patients

This study reveals that the incidence of postoperative frailty among BC patients was 29.37%, a finding consistent with that reported by Du (2024). However, this rate is lower than the 35.8% reported by Wang et al. (2024) in patients with extended survival, yet higher than the 15.4% preoperative frailty incidence documented by Lu et al. (2023) in elderly patients. These discrepancies may stem from differences in treatment stages among research populations. Wang et al. (2024) enrolled patients who had completed multimodal treatment, including surgery, chemotherapy, radiotherapy, and targeted therapy, whereas Lu et al. (2023) enrolled preoperative patients who had not yet received adjuvant therapies. The subjects of this study were postoperative BC patients who had either received or not received neoadjuvant therapy prior to surgery. The survey was conducted 3–5 days postoperatively, a time point at which patients had not yet begun postoperative adjuvant therapy. Therefore, this study primarily examines the impacts of the surgery itself and preoperative factors (including preoperative neoadjuvant therapy and patient-related factors) on postoperative frailty, and does not address the potential role of postoperative adjuvant therapy.

Unlike the BC surgeries examined in this study, thoracic or abdominal surgeries (such as those for lung or gastrointestinal cancers) are typically associated with greater tissue trauma, longer anesthesia times, and a higher risk of postoperative complications. Consequently, the incidence of postoperative frailty is often higher in these cases, and its impact on prognosis is more significant. Previous studies have shown that the incidence of postoperative frailty among elderly patients undergoing major abdominal surgery can exceed 40%, and it is closely associated with adverse outcomes such as postoperative delirium and prolonged hospital stays (Hewitt et al., 2018). Surgical procedures may cause physical stress and damage, potentially leading to declined physical function, increased nutritional consumption, and impaired immunity, thereby elevating frailty risk (Brouwers et al., 2016; Morgan et al., 2020). In contrast, BC surgery is relatively minimally invasive. However, it is worth noting that, in addition to coping with the physical trauma caused by surgery, BC patients must also confront the psychological impact of losing their female secondary sexual characteristics and the fear of the unknown associated with postoperative adjuvant therapy. Therefore, the mechanisms underlying postoperative frailty in these patients may involve both physiological and psychological pathways.

Meanwhile, frailty itself represents a critical determinant of health outcomes in BC patients, predicting postoperative complications, chemotherapy intolerance, disease progression, and mortality (Mandelblatt et al., 2017; Williams et al., 2019). Therefore, thorough analysis of influencing factors and the development of an accurate risk prediction model are particularly important. This model would assist healthcare professionals in early identification of high-risk patients, enable timely interventions to ameliorate frailty, facilitate recovery, and ultimately improve overall treatment outcomes and quality of life.

### Analysis of influencing factors for postoperative frailty in BC patients

#### Diabetes mellitus is a risk factor for postoperative frailty in BC patients

The results of this study showed that BC patients with a history of diabetes had a higher risk of postoperative frailty (OR = 5.24). This finding is consistent with the research results of Bu et al. (2023). The potential mechanisms underlying this association may be related to diabetes-induced sarcopenia, chronic inflammation, and complications involving peripheral nerves and blood vessels. Insulin resistance and hyperglycemia can inhibit protein synthesis in the body and reduce skeletal muscle energy metabolism, leading to decreased muscle mass and impaired muscle function. Notably, sarcopenia is recognized as a core pathological basis for frailty (Anagnostis et al., 2020; Bellary et al., 2021). One study indicated that the incidence of sarcopenia in diabetic patients is 2–3 times higher than that in non-diabetic patients (Qiao et al., 2021).

Additionally, diabetic patients often exhibit a state of chronic low-grade inflammation. Inflammatory factors can promote the development of frailty through multiple pathways and show a positive correlation with frailty (Sanz-Cánovas et al., 2022; Walker et al., 2019). For postoperative BC patients, the tumor itself and surgical trauma further activate the immune system, creating a “synergistic effect” with the chronic inflammatory state of diabetes and significantly increasing the systemic inflammatory burden.

Furthermore, diabetes-related complications such as peripheral neuropathy and microvascular dysfunction may exacerbate postoperative limb edema, pain, and limited mobility in BC patients. This reduction in physical activity further aggravates the degree of frailty (Sinclair, Abdelhafiz & Rodríguez-Mañas, 2017). Meanwhile, long-term insufficient nutrient intake also makes BC patients with diabetes more susceptible to frailty.

Therefore, it is recommended that clinical medical staff adopt comprehensive management strategies. These strategies include preoperative blood glucose monitoring, dietary guidance, nutritional assessment, and intervention. For patients with significant blood glucose fluctuations, delaying the operation may be considered. After surgery, monitoring of inflammatory markers, anti-inflammatory treatment when necessary, and regular screening and intervention for diabetes-related complications are essential to reduce the risk of postoperative frailty in BC patients.

#### Poor sleep quality is a risk factor for postoperative frailty in BC patients

The results of this study indicated that BC patients with poor sleep quality had a higher risk of postoperative frailty (moderately poor sleep: OR = 3.66; severely poor sleep: OR = 25.29). This finding is consistent with the research by Zhu et al. (2022). The underlying reasons may be linked to sleep disorder-induced dysregulation of the neuroendocrine system, immune dysregulation, and negative emotions (Bailur et al., 2017; Zhu et al., 2022). Sleep is a critical physiological process for the body’s self-repair, energy storage, immune regulation, and maintenance of neuroendocrine balance (Moreno-Tamayo et al., 2021). Long-term poor-quality sleep can lead to hyperactivity of the hypothalamic-pituitary-adrenal (HPA) axis, resulting in increased cortisol secretion. Sustained high cortisol levels can enhance protein catabolism, cause muscle atrophy and fat redistribution, and inhibit the activity and quantity of immune cells, thereby inducing chronic inflammation (Zhang et al., 2025). All these are core pathophysiological features of frailty (Gilmore et al., 2021; van Dalfsen & Markus, 2018).

In addition, poor sleep quality can indirectly promote the development of frailty by affecting the psychological state of BC patients after surgery (Liu et al., 2021c). Postoperative BC patients often bear a heavy psychological burden due to concerns about postoperative recovery, fear of tumor recurrence, anxiety about adjuvant therapy, and loss of physical secondary sexual characteristics (King et al., 2024). These negative emotions and sleep disorders often form a vicious cycle, leading to physical and psychological frailty (Bao et al., 2017; Liu et al., 2021a).

Therefore, early identification and intervention of sleep disorders are crucial in the management of postoperative frailty in BC patients. It is recommended that clinical healthcare professionals incorporate sleep assessment into routine practice. For patients with sleep disorders or high-risk groups, comprehensive measures, including cognitive behavioral therapy, sleep hygiene education, and medication intervention when necessary, should be implemented promptly to reduce the incidence of frailty and improve patients’ health outcomes.

#### Higher total cholesterol is a protective factor for postoperative frailty in BC patients

The results of this study showed that BC patients with higher cholesterol levels had a lower risk of postoperative frailty (OR = 0.60). This finding is consistent with the research by Yin et al. (2023). The potential reason may be related to cholesterol’s role as a critical precursor for synthesizing steroid hormones (such as testosterone, estrogen, progesterone, and growth hormone), which are involved in muscle growth (Liu et al., 2021b). Sarcopenia, as a key pathophysiological factor in the aging process, accelerates the progression of frailty (Hwang et al., 2015). Maintaining an appropriate cholesterol level helps reduce the incidence of sarcopenia in older adults, thereby lowering the risk of frailty (Gu et al., 2023). Additionally, cholesterol plays a crucial role in the formation of mature immune synapses (Huang, Song & Xu, 2020); higher cholesterol levels may enhance the body’s immune function, further reducing the risk of frailty in postoperative BC patients.

However, it is important to emphasize that the protective effect of “higher total cholesterol” observed in this study was found in patients whose total cholesterol levels were normal or slightly elevated; it does not support the notion that cholesterol levels can rise indefinitely. Excessively high cholesterol may promote the proliferation of estrogen receptor-positive breast cancer cells through its metabolite 27-hydroxycholesterol (Nazih & Bard, 2020). Therefore, dietary cholesterol intake must be moderate. It should meet the body’s normal physiological needs and maintain health, while avoiding excessive levels that may have adverse effects on BC patients.

It is recommended that clinical healthcare professionals improve patients’ understanding of cholesterol’s important role in muscle synthesis and maintenance. They should also collaborate with dietitians to develop personalized dietary guidance plans for BC patients. These plans should ensure adequate intake of high-quality protein to maintain muscle mass and appropriately increase consumption of foods rich in unsaturated fatty acids (such as nuts and olive oil), thereby reducing the risk of frailty.

#### Having children as primary caregivers is a protective factor for postoperative frailty in BC patients

The results of this study indicated that BC patients with adult children as their primary caregivers had a lower risk of postoperative frailty (OR = 0.36). The potential reason may be that BC patients after surgery have a significantly increased demand for social support due to physical trauma and psychological stress. As providers of intergenerational support in their parents’ social support network, adult children can better offer emotional support, daily care, and financial assistance to patients. This improves the patients’ social support level and reduces the incidence of frailty (Fan et al., 2021; Shu et al., 2021). Intergenerational support refers to the process of resource exchange between adult children and their parents within a family, and it is a key component of parents’ social support (Li, Jiang & Zhang, 2019).

Firstly, as primary caregivers, adult children can provide better emotional support. This helps patients enhance their ability to cope with negative life events, increase positive emotional experiences, alleviate physical and mental distress, and effectively delay the onset of frailty (Luger et al., 2016; Maltby et al., 2020). Secondly, adult children usually have stronger learning abilities and information-acquisition skills, enabling them to understand and implement the rehabilitation guidance plans from the medical team more effectively. Additionally, adult children as primary caregivers can provide more stable financial security for patients, ensuring they have timely and effective access to medical resources and rehabilitation services. This avoids insufficient treatment or rehabilitation measures due to financial constraints, thereby reducing the risk of frailty to a certain extent (Shu et al., 2021).

Therefore, in the clinical practice for postoperative BC patients, clinical healthcare professionals should not only focus on patients’ physical needs but also fully mobilize their social support systems. By strengthening communication with patients’ family members, encouraging family members to participate in the patients’ diagnosis, treatment, and rehabilitation processes, and helping family members better understand the patients’ physical and psychological needs, more targeted support can be provided.

#### Urban Employee Basic Medical Insurance (UEBMI) is a protective factor for postoperative frailty in BC patients

The results of this study showed that BC patients with UEBMI had a lower risk of postoperative frailty (OR = 0.29). This finding highlights the important role of the social medical security system in the treatment and postoperative rehabilitation of BC patients. UEBMI is one of the main components of China’s social medical insurance system. It primarily covers active and retired employees of government agencies, enterprises, and public institutions, and typically features higher contribution rates and reimbursement ratios (Liu, Liu & He, 2023). Compared to Urban Resident Basic Medical Insurance (URBMI) or the New Rural Cooperative Medical Scheme (NCMS), it offers a higher level of coverage. In this study, patients enrolled in UEBMI had a lower risk of postoperative frailty, which may be attributed to their higher level of coverage, lower financial burden associated with medical care, and better access to medical resources. Research has found that social medical security is an important factor contributing to differences in frailty indices (Chen, 2024). Especially in rural areas, the reimbursement rate of the NCMS is relatively low, leading to a heavier economic burden of illness for rural patients (Wang, 2020). In contrast, employee medical insurance reduces patients’ frailty risk through multiple pathways, including economic burden reduction, improved accessibility to medical resources, and promotion of healthy behaviors.

BC patients generally face varying degrees of economic burden due to complex treatment plans and long disease courses, resulting in a higher demand for social medical security (Irwin et al., 2014; Jagsi et al., 2014). Therefore, further improving the social medical security system, especially expanding the coverage and increasing the reimbursement rate of medical insurance in rural areas, is of great significance for improving the quality of life of postoperative BC patients and reducing their frailty risk.

At the same time, UEBMI coverage also reflects patients’ socioeconomic status to some extent, as stable employment and higher income are often prerequisites for insurance enrollment. Therefore, the protective effect of UEBMI against postoperative frailty can be partially explained by its association with socioeconomic status (Hanlon et al., 2024). In this study, monthly per capita household income was associated with frailty in univariate analysis but did not emerge as an independent predictor in multivariate analysis. This may be attributed to the limited sample size or multicollinearity between income and other variables (such as health insurance type and caregivers). Income levels may indirectly influence the onset of frailty by affecting access to medical resources, nutritional support, and rehabilitation conditions. Future studies should further employ multidimensional socioeconomic status indicators to explore in depth the mechanisms underlying their role in the development of postoperative frailty. Additionally, future studies should pay more attention to the actual implementation effects of different types of medical insurance policies and explore more targeted intervention measures, aiming to provide better full-cycle health management services for BC patients.

#### A higher BMI is a protective factor for postoperative frailty in BC patients

The results of this study showed that BC patients with higher BMI had a lower risk of postoperative frailty (OR = 0.86). Zhou et al. (2025) in their study on frailty in patients after acute myocardial infarction intervention, found that decreased BMI increased the risk of frailty. They speculated that BMI is positively correlated with skeletal muscle mass, which is consistent with the results of this study. Yu et al. (2024) also found a positive correlation between BMI and muscle mass, meaning higher BMI indicates higher muscle mass. Yu et al. (2025) discovered that higher BMI is a protective factor against osteoarthritis (OS), further supporting this view. Skeletal muscle mass and muscle strength are key indicators of frailty (Hamid et al., 2024). Based on this, it can be further inferred that higher BMI may be a protective factor against frailty.

Previous large-scale cross-sectional studies have shown that a lower BMI range (particularly values below 18.5 kg/m^2^) is closely associated with a higher risk of low muscle mass (Xie et al., 2025). Conversely, a higher BMI typically indicates adequate energy reserves and relatively greater skeletal muscle mass, which helps the body better cope with the high metabolic demands following surgery, prevents rapid weight loss and muscle wasting, and reduces the risk of frailty. At the same time, BC patients often experience weight and muscle loss after surgery due to insufficient nutrient intake, metabolic disorders, and treatment-related side effects (such as nausea, vomiting, loss of appetite), which increase the risk of postoperative frailty. Higher BMI helps increase energy reserves, better cope with the high metabolic demand of the body after surgery, prevent rapid weight loss and muscle wasting, and thus reduce the risk of frailty. However, some studies (Wei et al., 2023) have found a U-shaped relationship between BMI and survival, with the lowest risk observed when BMI is around 23 kg/m^2^. Both excessively high and low BMI can increase the risk of frailty (Sun, Xia & He, 2024; Yuan, Chang & Wang, 2021). Therefore, maintaining BMI within the normal range is recommended to be more beneficial for frailty prevention.

In clinical practice, healthcare professionals should develop personalized nutrition and exercise plans tailored to each patient’s specific circumstances to maintain an appropriate BMI level. They should conduct comprehensive assessments that take into account both muscle mass and body fat percentage, rather than focusing solely on increasing BMI. For breast cancer patients with a low BMI, it is necessary to monitor weight regularly and develop a reasonable nutritional plan to increase both weight and muscle mass, thereby meeting the body’s postoperative metabolic needs and further reducing the risk of frailty.

#### Higher exercise frequency (≥3 times/week) is a protective factor for postoperative frailty in BC patients

The results of this study showed that BC patients with higher exercise frequency (≥3 times/week) had a lower risk of postoperative frailty (OR = 0.20). This finding is consistent with the research by Zhou et al. (2020) and aligns with the conclusion of Chen et al. (2024) regarding the dose–response relationship between exercise and frailty. The potential reasons may be as follows: Firstly, sarcopenia is a key pathological basis for the development of frailty (Bellieni et al., 2021). Regular high-frequency exercise can enhance muscle strength, improve physical fitness, and boost the body’s immune function, thereby effectively reducing the risk of frailty. Progressive resistance training (such as dumbbell and equipment-based strength training), moderate aerobic exercise (such as brisk walking and jogging), and traditional Chinese medicine exercise therapies (such as Tai Chi, Yijinjing, and Wuqinxi) all improve immune function, increase muscle strength, and reduce visceral fat content, ultimately alleviating patients’ frailty (Chen et al., 2020; Rodrigues et al., 2022). Secondly, obese patients are more prone to postoperative frailty due to increased metabolic burden and higher risk of complications (Feng et al., 2021). Regular high-frequency exercise helps maintain a healthy body weight, thus lowering the risk of frailty. Thirdly, regular high-frequency exercise significantly improves mental state, effectively relieves anxiety and depression, and reduces cancer-related fatigue (Zhou et al., 2022), which in turn reduces the risk of frailty. Finally, exercise intervention can improve attention, processing speed, and executive function in patients with cognitive frailty, and may delay or even reverse cognitive frailty (Katayama et al., 2021).

Therefore, clinical healthcare professionals should provide personalized exercise guidance to postoperative BC patients on the premise of ensuring exercise safety. They should encourage and guide patients to develop regular exercise habits in daily life, and ensure long-term adherence through regular follow-ups and family supervision. This will enhance patients’ physical tolerance and further reduce the risk of frailty.

#### Undergoing breast-conserving surgery is a protective factor for postoperative frailty in BC patients

The results of this study indicated that BC patients undergoing breast-conserving surgery had a lower risk of postoperative frailty (OR = 0.33). The underlying mechanisms can be explained from both physiological and psychological perspectives. From a physiological perspective, modified radical mastectomy requires complete resection of the breast and axillary lymph node dissection, resulting in extensive tissue damage. In contrast, breast-conserving surgery only removes local lesions and often replaces axillary lymph node dissection with less invasive sentinel lymph node biopsy, leading to a milder systemic inflammatory response postoperatively (Radin et al., 2022). Patients can resume daily activities earlier after surgery, which effectively preserves skeletal muscle reserves and reduces physiological triggers of frailty. Furthermore, differences in physical trauma further lead to divergent psychological status among patients. Breast loss acts as a major stressor triggering body image disturbance, anxiety and depression (Sun et al., 2020). Breast-conserving surgery fully preserves breast contour and can significantly improve patients’ postoperative psychological well-being. Large-sample follow-up studies have confirmed (Huang et al., 2026) that patients who underwent breast-conserving surgery achieve higher scores in breast satisfaction, psychological well-being and sexual function at 6 months postoperatively compared with those undergoing mastectomy, with the detection rate of anxiety and depression reduced by more than 40%. Reduction of negative emotions can suppress excessive activation of the hypothalamic-pituitary-adrenal (HPA) axis, prevent cortisol-induced sustained muscle breakdown and immune dysfunction, and block the progression of frailty triggered by the bidirectional interaction between psychological and physical conditions (Alhazzaa et al., 2026).

Although implant-based breast reconstruction can restore breast appearance, this procedure involves mastectomy followed by prosthesis implantation. The dual surgical procedures cause greater trauma; postoperative complications such as traction pain and capsular contracture continuously increase physical burden, offsetting the psychological benefits brought by breast reconstruction (Radin et al., 2022). In addition, only 27 patients received implant-based breast reconstruction in this study. The small sample size of this subgroup led to insufficient statistical power, so no significant protective effect of this surgical method against frailty was observed.

In conclusion, our observational findings suggest that breast-conserving surgery may be associated with lower risk of postoperative frailty among early-stage breast cancer patients meeting surgical eligibility criteria (provided oncologic safety can be guaranteed). This observed association warrants further investigation in future studies. For patients undergoing modified radical mastectomy or implant-based breast reconstruction, upper limb rehabilitation, psychological counseling, nutritional guidance and regular exercise interventions should be implemented simultaneously to comprehensively lower the incidence of frailty.

### The nomogram model had good predictive performance in predicting postoperative frailty risk in BC patients

The nomogram model constructed in this study demonstrated significant predictive value for assessing postoperative frailty risk in BC patients. The AUC was 0.813, with a specificity of 0.792, a sensitivity of 0.726, an optimal cut-off value of 0.327. And a Youden index of 0.518, and after rigorous internal validation, the AUC value of each fold was greater than 0.7, with a mean AUC of 0.791. This result indicates that the nomogram not only performs well in distinguishing patients at different risk levels but also exhibits high reliability in predictive stability. Furthermore, the Hosmer-Lemeshow test results showed a *χ*^2^ value of 4.436 (*P* = 0.816). This statistical outcome confirms that the predicted probability of frailty estimated by the model is highly consistent with the actual observed frailty occurrence, reflecting the model’s good consistency and accuracy.

In clinical practice, this nomogram provides healthcare professionals with a simple and efficient scoring tool. With this tool, medical staff can quickly assess the frailty risk of BC patients after surgery. The assessment process is not only easy to operate but also enables the timely and accurate identification of patients at high risk of frailty. Based on this, healthcare providers can develop and implement targeted, personalized interventions to proactively control potential risk factors, thereby effectively reducing the incidence of postoperative frailty in BC patients and improving their overall rehabilitation outcomes and quality of life. It should be noted that some variables in this study (*e.g.*, marital status subgroups) , showed relatively wide confidence intervals. This suggests potential instability in effect estimates due to limited sample size and uneven subgroup distribution. Therefore, these findings should be interpreted with caution, and further validation in larger, multicenter cohorts is warranted.In addition, some variables that were statistically significant in univariate analysis, such as age (*P* = 0.011), were not retained in the final multivariable model. This may be attributed to LASSO variable selection, multicollinearity between variables, or the effect of age being explained by other factors such as sleep quality, BMI, and exercise frequency. Their exclusion does not negate their potential clinical relevance but rather reflects the statistical preference for a more compact predictive model.

## Summary

In summary, BC patients face a relatively high risk of frailty after surgery. Risk factors for postoperative frailty include comorbid diabetes and poor sleep quality. In contrast, protective factors include higher total cholesterol levels, having adult children as primary caregivers, UEBMI, higher BMI, higher frequency of exercise (≥3 times/week), and undergoing breast conserving surgery. The nomogram constructed based on these factors exhibits high predictive value and provides practical guidance for healthcare professionals in clinical practice.

However, this study has certain limitations: It is a single-center study, with all participants recruited from one tertiary general hospital, leading to a relatively small sample size. The number of included predictive factors is limited. Future studies should conduct external validation using multi-center, large-sample data and incorporate more relevant predictive factors to further explore the risk factors for postoperative frailty in BC patients. Furthermore, due to differences in healthcare systems, dietary habits, health cultures, and social security policies, the generalizability of this predictive model across different countries, healthcare systems, and cultural contexts requires further validation. However, the risk and protective factors identified in this study, as well as the assessment tools used, can serve as a valuable reference for developing similar frailty prediction models in other regions or countries.

##  Supplemental Information

10.7717/peerj.21733/supp-1 Supplemental Information 1 Raw data

10.7717/peerj.21733/supp-2 Supplemental Information 2 Assignment Method for Categorical Variables

10.7717/peerj.21733/supp-3 Supplemental Information 3 STROBE Statement

## Additional Information and Declarations

### Data Availability

The following information was supplied regarding data availability: The raw data is available in the Supplemental Files.
