# Supplementary material for: Development and validation of a risk prediction model for postoperative frailty in breast cancer patients
Source: PeerJ. 2026 Sep 22;14:e21733. doi: 10.7717/peerj.21733 (PMC13614270; doi:10.7717/peerj.21733)
Supplement: Supplementary file 1 [file peerj-14-21733-s002.docx]

Codebook. Assignment Method for Categorical Variables

| **Independent Variable** | **Assignment Method** |
| --- | --- |
| Marital Status | Unmarried=1, Married=2,Divorced/Widowed=3 |
| Primary caregiver | Spouse=1,Parents=2,Children=3,Other=4 |
| Education level | Primary school and below=1, Junior school and below=2,College and above=3 |
| Occupation | Employed=1, Retirement=2,Unemployed or other=3 |
| Medicare types | Resident insurance=1,employee medical insurance=2 |
| Residence | Rural areas=1,Towns=2 |
| Monthly household income per capita | <1000=1, 1000-5000=2,>5000=3 |
| Complicated with diabetes,  Complicated with hypertension,  Complicated with cerebral infarction | Yes=1,No=0 |
| Surgical method | Modified Radical Mastectomy=1,  Breast-Conserving Surgery=2,  Implant-Based Reconstruction=3 |
| Pathological Type | Carcinoma in situ=1, Invasive carcinoma=2 |
| Neoadjuvant chemotherapy,  Postoperative gastrointestinal reaction,  Oral mucositis | Yes=1，No=0 |
| Exercise Frequency | None=1,<3 times/week=2,>3 times/week=3 |
| PSQI | good sleep quality=1,poor sleep quality=2,very poor sleep quality=3 |
| TFI | Frailty=1,Non-frailty=0 |
